# Associations between quantitative measures of mammographic density and terminal ductal lobular unit involution in Chinese breast cancer patients

**DOI:** 10.1186/s13058-024-01856-z

**Published:** 2024-07-15

**Authors:** Waruiru Mburu, Changyuan Guo, Yuan Tian, Hela Koka, Sheng Fu, Ning Lu, Erni Li, Jing Li, Renata Cora, Ariane Chan, Jennifer L. Guida, Hyuna Sung, Gretchen L. Gierach, Mustapha Abubakar, Kai Yu, Xiaohong R. Yang

**Affiliations:** 1 Division of Cancer Epidemiology and Genetics, DHHS, National Cancer Institute, NIH, 9609 Medical Center Drive, Bethesda, MD 20892-9761 USA; 2 National Cancer Center/National Clinical Research Center for Cancer/Cancer Hospital, Chinese Academy of Medical Sciences and Peking Union Medical College, Beijing, 100021 China; 3 Volpara Health Technologies Ltd, Wellington, New Zealand; 4 Division of Cancer Control and Population Sciences, DHHS, National Cancer Institute, NIH, 9609 Medical Center Drive, Bethesda, MD 20892-9761 USA; 5 Surveillance and Health Equity Science, American Cancer Society, Atlanta, GA 30303 USA; 6 Institute of Environmental Science and Research, Porirua, GA 5022 New Zealand

**Keywords:** Mammographic density, TDLU involution, Chinese breast cancer patients, Volpara

## Abstract

**Background:**

Higher mammographic density (MD), a radiological measure of the proportion of fibroglandular tissue in the breast, and lower terminal duct lobular unit (TDLU) involution, a histological measure of the amount of epithelial tissue in the breast, are independent breast cancer risk factors. Previous studies among predominantly white women have associated reduced TDLU involution with higher MD.

**Methods:**

In this cohort of 611 invasive breast cancer patients (ages 23–91 years [58.4% ≥ 50 years]) from China, where breast cancer incidence rates are lower and the prevalence of dense breasts is higher compared with Western countries, we examined the associations between TDLU involution assessed in tumor-adjacent normal breast tissue and quantitative MD assessed in the contralateral breast obtained from the VolparaDensity software. Associations were estimated using generalized linear models with MD measures as the outcome variables (log-transformed), TDLU measures as explanatory variables (categorized into quartiles or tertiles), and adjusted for age, body mass index, parity, age at menarche and breast cancer subtype.

**Results:**

We found that, among all women, percent dense volume (PDV) was positively associated with TDLU count (highest tertile vs. zero: Exp^beta^ = 1.28, 95% confidence interval [CI] 1.08–1.51, p_trend_ =  < .0001), TDLU span (highest vs. lowest tertile: Exp^beta^ = 1.23, 95% CI 1.11–1.37, p_trend_ =  < .0001) and acini count/TDLU (highest vs. lowest tertile: Exp^beta^ = 1.22, 95% CI 1.09–1.37, p_trend_ = 0.0005), while non-dense volume (NDV) was inversely associated with these measures. Similar trend was observed for absolute dense volume (ADV) after the adjustment of total breast volume, although the associations for ADV were in general weaker than those for PDV. The MD-TDLU associations were generally more pronounced among breast cancer patients ≥ 50 years and those with luminal A tumors compared with patients < 50 years and with luminal B tumors.

**Conclusions:**

Our findings based on quantitative MD and TDLU involution measures among Chinese breast cancer patients are largely consistent with those reported in Western populations and may provide additional insights into the complexity of the relationship, which varies by age, and possibly breast cancer subtype.

**Supplementary Information:**

The online version contains supplementary material available at 10.1186/s13058-024-01856-z.

## Background

Mammographic density (MD) is a radiographic appearance of stroma and epithelium (fibroglandular) breast tissues [1]. High MD is an established risk factor for breast cancer—women with a percent density of 75% or more have 4.64 greater risk of breast cancer compared to women with less than 5% density [2]. Although underlying mechanisms remain unclear, previous studies suggest that MD-associated breast cancer risk may partially be mediated through the abundance of epithelial cells that are susceptible to carcinogenesis [3]. Terminal duct lobular units (TDLUs) are milk producing epithelial structures within the breast where most breast carcinomas and their precursors originate [4, 5]. Age-related TDLU involution is characterized by a reduction in the number and size of TDLUs and their substructures (acini) [5]. Reduced TDLU involution has been associated with a higher risk for breast cancer in studies of women with benign breast disease (BBD) [6, 7]. Both MD and TDLU involution have shown variations by breast cancer subtype among breast cancer patients. We previously showed that high MD was associated with HER2-enriched and luminal B breast cancer subtypes among Chinese breast cancer patients [8, 9], and that reduced TDLU involution was associated with basal-like or triple-negative subtypes among European, Black, and Chinese breast cancer patients [10–12]. These data highlight the potential value of these breast tissue composition measures as markers of etiologic and molecular heterogeneity of breast cancer. Studies have also shown racial and ethnic variations in MD, with Asian women having higher MD [13, 14]. Using TDLU data collected from > 2000 healthy women who donated breast tissue to the Komen tissue bank in the United States, we previously found that women of African ancestry were more likely than women of European ancestry to have increased TDLU number, density, and size (indicating less extent of TDLU involution), whereas women of East Asian ancestry tended to have increased involution, after adjustment for potential confounding factors [15]. The heightened MD contrasted with increased TDLU involution in Asian women is intriguing, prompting inquiry into whether this relationship between TDLU involution and MD is distinct among Asian women.

Previous studies evaluating the association between TDLU involution and MD have generated mixed results. While most studies including ours have shown an inverse relationships between MD and TDLU involution, i.e., reduced TDLU involution associated with higher MD [3, 16–18], a study of breast cancer patients in the Multiethnic Cohort (MEC, comprising 33.5% Caucasian, 46.2% Japanese American, 11.0% Native Hawaiian, and 9.3% other) showed that greater TDLU involution was associated with higher absolute dense area, but not with percent density [19]. The conflicting results may be due to differences in study populations (e.g., breast cancer vs. BBD, race/ethnicity, etc.), MD/TDLU assessment measures (categorical vs. quantitative, percent vs. absolute MD, or area-based vs. volumetric MD), and sample sizes. To clarify the relationship between MD and TDLU involution, we collected objective, quantitative MD and TDLU measures in a large cohort of Chinese breast cancer patients, which allowed us to examine the MD/TDLU relationship by age (< 50 and ≥ 50 years) and breast cancer subtype. In particular, we used volumetric MD measurements, which could take into account tissue depth.

## Methods

### Study population

We used data from a cohort of 1,549 invasive breast cancer patients who were diagnosed and treated at the Cancer Hospital, Chinese Academy of Medical Sciences (CHCAMS), Beijing, China, between March 2016 and July 2017, as previously described in detail [20]. In brief, these patients had a confirmed invasive breast cancer diagnosis, complete immunohistochemical (IHC) marker status for breast cancer subtype definition, quantitative MD assessments, and breast cancer risk factor data. None of the patients had received neoadjuvant treatment prior to surgery. In the current analysis, we selected patients for whom adjacent normal tissue blocks from surgical specimens were available for TDLU evaluations. After excluding patients with cancer (including ductal carcinoma in situ) present in adjacent normal blocks for TDLU evaluation, the current analysis included 611 patients. These subjects did not overlap with those included in our previous evaluation of MD-TDLU relationship based on the Breast Imaging Reporting and Data System (BI-RADS) density classification in a much smaller set of CHCAMS patients (n = 144) who were diagnosed between 2009 and 2012 [18]. We compared the 611 patients to the 1,016 that were not included, and they were similar except the 611 patients included in the current analysis were more likely to have breastfed and had slightly higher frequency of Luminal B and triple negative breast cancer subtypes (Supplementary Table 1). The project was approved by the CHCAMS ethics committee and was exempted from review by the Office for Human Research Protections at the National Institutes of Health because it did not involve interaction with human subjects and/or use of personal identifying information (exempt no. 11751).

Breast cancer risk factors were extracted from medical records, and they included age at diagnosis, family history of breast cancer, age at menarche, parity, breastfeeding, and body mass index (BMI). Clinical characteristics and IHC marker status were extracted from pathology reports. Determination of marker positivity was previously described in detail [20]. Molecular subtypes were defined as follows: luminal A: ER+ and PR+, HER2−, and low Ki-67 (< 25%) or histologic grade (I or II); luminal B/HER2+: ER+ or PR+, and HER2+; luminal B/HER2−: ER+ or PR+, HER2−, and high Ki-67 (≥ 25%) or histologic grade (III); HER2-enriched (HER2-E): ER−, PR−, and HER2+; and triple-negative (TN): ER−, PR−, and HER2−. Due to small sample sizes of some subtypes, for the main analysis, we combined the breast cancer subtypes into three groups: luminal A, luminal B (luminal B/HER2− and luminal B/HER2+), and non-luminal (HER2-E and TN).

### TDLU assessment

Histologic assessment of TDLU involution, as previously described in detail [10], was performed by a single trained cytotechnologist (Renata Cora), who was blinded to mammographic density, patient and tumor data. Briefly, hematoxylin and eosin staining H&E-stained tissue sections from adjacent normal tissue blocks were scanned and digitally annotated for three TDLU measures: TDLU count per unit area (count/100 mm^2^), TDLU span (measured with an electronic ruler in micrometers), and acini count per TDLU. Higher level of each of these measures reflects lower level of TDLU involution [21]. For women with TDLUs observed (93.9%), up to 10 TDLUs were evaluated for acini count per TDLU (categories: 2–10, 11–20, 21–30, 31–50, 51–100, and ≥ 100) and TDLU span per slide, as previously described [11]. For TDLU span and acini counts per TDLU, we evaluated mean, median and maximum values across multiple TDLUs measured for each woman. Multiple adjacent normal tissue blocks within a single patient were available for a subset of patients (N = 142), which were used to assess intra-person variability. Among patients with multiple tissue blocks, the intraclass correlation coefficients (ICCs) were equivalent for the mean and median values for acini count/TDLU (0.60 and 0.60, respectively) and similar for TDLU span (0.53, 0.54, respectively), and were higher than maximum acini count/TDLU (0.54) and TDLU span (0.39) (see Supplementary Table 2). We therefore chose mean values across TDLUs within a patient for these variables in the primary analyses.

### MD assessment

A fully automated quantitative density assessment tool, VolparaDensity software 5th edition** (**Volpara Health Technologies, Wellington, New Zealand) was used to estimate total breast and absolute dense (fibroglandular tissue) volume in cm^3^ [22]. Percent dense volume (PDV) was calculated by dividing absolute dense volume (ADV) by the total volume of the breast. Non-dense volume (NDV) was the difference between total breast volume (TBV) and ADV. The correlations of MD between the left and right breast were very high (87–95%) for all MD measures. Therefore, we used MD values from the unaffected contralateral breast in all downstream analyses. We also conducted a sensitivity analysis using MD values in the diseased breast when assessing TDLU-MD relationships and the results were very similar.

### Statistical analysis

One-way analysis of variance was used to assess the associations between continuous MD measures (PDV, ADV, and NDV) and patient characteristics which included: age at diagnosis (23–39, 40–49, 50–59, 60–69 and 70 + years), BMI (< 23, 23–24.9, 25+), age at menarche (≤ 12, 13–14 and 15 + years), family history of breast cancer (yes vs no), parity (nulliparous vs parous) and breast cancer subtype (luminal A, luminal B, HER2-enriched and triple negative). Spearman rank correlations were used to assess correlations between continuous metrics of TDLU involution and MD measures and patient characteristics (age at diagnosis, BMI, age at menarche and parity) stratified by age (< 50 and ≥ 50 years), as proxy for menopausal status.

In primary analyses, we used generalized linear models to examine the associations between TDLU involution and MD. Quantitative MD measures (PDV, ADV, and NDV) were log-transformed to mitigate their skewness. Three separate models were fitted with PDV, ADV, and NDV (log-transformed) as the outcome variables and TDLU involution measures (TDLU count/100 mm^2^; median TDLU span; and median acini/TDLU) as explanatory variables (categorized into three or four levels). For women with observable TDLUs, overall and age-group specific tertiles of median TDLU span and median acini count/TDLU were calculated. All models were adjusted for age at diagnosis (23–39, 40–49, 50–59, 60–69, 70+), BMI (< 23, 23–24.9, 25+), parity (nulliparous, parous), and age at menarche (≤ 12, 13–14, 15+). Since breast cancer subtype was previously associated with both MD and TDLU variables in our previous analyses of Chinese breast cancer patient data collected from the same hospital [8–10], we also included breast cancer subtype (luminal A, luminal B, non-luminal) as a covariate in the model. Models for ADV were further adjusted for total breast volume (TBV). Participants with missing data on certain covariates were included in the analysis using separate missing data categories. β coefficients and 95% confidence intervals (CIs) from the regression models were evaluated and back transformed for easier interpretation. P_trend_-values were obtained by treating the categorical TDLU variables as ordinal variables in trend analyses. We further stratified the results to examine whether TDLU involution-MD associations differed by age (< 50 and ≥ 50 years) and breast cancer subtype (Luminal A, Luminal B and non-Luminal) [10, 11, 20]. To maximize all TDLU span measurements across different TDLUs and multiple blocks within a patient, in a separate analysis, we analyzed all individual data points for TDLU span (including those obtained from multiple tissue blocks within a patient) rather than using the median value, by applying generalized estimating equations (GEE) models to account for the lack of independence of repeated TDLU span measures. In the GEE model, we modeled TDLU span (continuous variable) as the outcome variable and PDV as explanatory variable. Since the GEE model works better with continuous variables, we were not able to use this approach to analyze the number of acini per TDLU.

All analyses were conducted using SAS (version 9.4; SAS institute Inc., Cary, NC) and R (version 4.1.1). All statistical tests were two-sided, and the level of significance set at *p* < 0.05.

## Results

### Participant characteristics and distributions of TDLU involution and MD measures

**Fig. 1:**
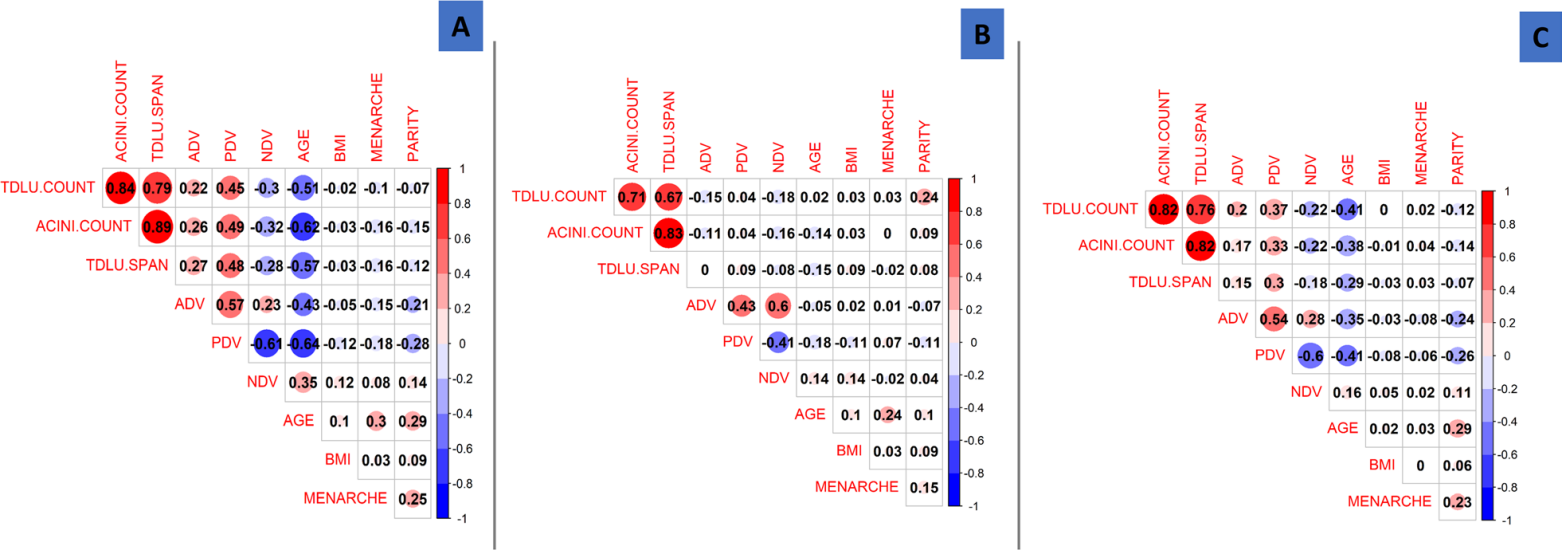
Correlation between terminal ductal lobular unit (TDLU) measures, mammographic density, and patient characteristics in 611 Chinese breast cancer patients. **A** Overall; **B** < 50 years; **C** >  = 50 years. Spearman rank correlations were used to assess correlations. Red: positive correlation; Blue: negative correlation. The size of the circle reflects the level of statistical significance

Table 1 shows the distributions of patient demographic and clinical characteristics and MD measures. The mean age (standard deviation [SD]) and BMI (SD) were 52.7 (11.3) years and 24.4 (4.2) kg/m^2^, respectively. Luminal B was the most common breast cancer subtype (44.8%). As expected, age was negatively associated with percent dense volume (PDV) and absolute dense volume (ADV), and positively associated with non-dense volume (NDV) (all *p*-values < 0.0001). Younger age at menarche (*p* = 0.02) was associated with higher ADV, while parity (*p* = 0.02) and high BMI (*p* = 0.04) were associated with lower PDV. Consistent with what was previously reported in this population [20], luminal B and HER2-enriched patients were more likely to have higher ADV, as compared to other subtypes (Table 1). In general, the correlations between MD measures and patient characteristics showed similar patterns among younger (< 50 years) and older (≥ 50 years) women, although they were more pronounced among older than younger women (Fig. 1). Overall, TDLU count/100 mm^2^, median TDLU span and median acini count/TDLU were highly correlated with each other (r^2^ = 0.79–0.89, *p* < 0.0001) and inversely correlated with age, age at menarche and parity (see Fig. 1, Supplementary Table 3). Interestingly, the associations between TDLU measures and parity appeared to vary by age, with positive correlations seen among younger women and negative correlations observed among older women (Fig. 1). Patients with Luminal B and Triple Negative Breast Cancer (TNBC) tumors were more likely to have greater TDLU counts and sizes compared to other subtypes, although the differences were not statistically significant (see Supplementary Table 3).

**Table 1:** Patient characteristics and distribution of mammographic density by patient characteristics

Characteristic			Percent dense volume (PDV)			Absolute dense volume (ADV)			Non Dense Volume (NDV)		
	N = 611	%	Mean	SD	*p* Value^a^	Mean	SD	*p* Value^a^	Mean	SD	*p* Value^a^
*Age at diagnosis, years*											
Mean, SD	52.72	11.29									
23–39	63	10.31	20.01	6.67	** < 0.0001**	69.95	42.96	** < 0.0001**	292.61	161.98	** < 0.0001**
40–49	191	31.26	18.12	6.37		69.82	41.48		332.33	170.60	
50–59	191	31.26	11.48	6.07		48.14	24.03		425.83	207.80	
60–69	121	19.80	9.13	5.79		39.81	20.91		459.45	219.82	
70 +	45	7.36	7.74	3.11		32.33	16.10		429.70	220.91	
*Body mass index kg/m*^*2*^											
Mean, SD	24.39	4.17									
< 23	219	35.84	14.72	7.87	**0.04**	54.72	32.07	0.42	373.80	227.22	0.26
23–24.9	140	22.91	13.69	7.44		58.66	39.70		409.62	200.59	
25 +	214	35.02	12.73	6.68		52.29	35.11		396.67	184.53	
Missing	38	6.22	14.74	7.49		53.05	27.02		352.26	169.34	
*Family history of breast cancer*											
Yes	56	9.17	12.99	6.90	0.71	53.46	26.83	0.90	420.16	249.37	0.47
No	476	77.91	13.88	7.42		55.00	35.63		387.08	197.51	
Missing	79	12.93	13.70	7.47		53.37	34.45		377.26	206.63	
*Age at menarche, years*											
≤ 12	66	10.80	15.44	7.50	0.09	65.84	41.90	**0.02**	400.56	214.27	0.59
13–14	184	30.11	14.28	7.36		56.49	38.81		385.96	225.22	
15 +	280	45.83	13.08	7.30		51.34	29.20		395.61	190.02	
Missing	81	13.26	13.61	7.38		52.27	34.07		361.90	188.13	
*Parity*											
Nulliparous	18	2.95	18.48	7.60	**0.02**	70.71	44.13	0.13	317.25	153.59	0.11
Parous	504	82.49	13.53	7.32		53.80	33.10		396.25	207.20	
Missing	89	14.57	14.28	7.40		56.27	40.96		360.14	188.05	
*Breastfeeding*											
Yes	453	74.14	13.63	7.41	0.56	54.00	34.25	0.27	394.31	207.43	0.44
No	71	11.62	14.67	7.47		60.89	40.02		383.99	195.07	
Missing	87	14.24	13.79	7.13		52.85	32.39		363.42	190.65	
*Breast cancer subtype*											
Luminal A	173	28.31	13.48	7.64	0.85	50.95	27.39	**0.03**	389.87	216.46	0.14
Luminal B	274	44.84	13.95	7.32		59.24	39.01		404.97	203.05	
HER 2 Enriched	37	6.06	13.64	7.29		57.43	35.43		388.98	162.20	
Triple Negative	76	12.44	13.34	7.11		50.59	37.68		369.97	205.61	
Missing	51	8.35	14.64	7.42		46.44	22.80		325.83	178.62	

Significant results based on *p* < 0.05

^a^*p* value was calculated using one way analysis of variance

### Associations between TDLU involution measures and MD measures

**Fig. 2:**
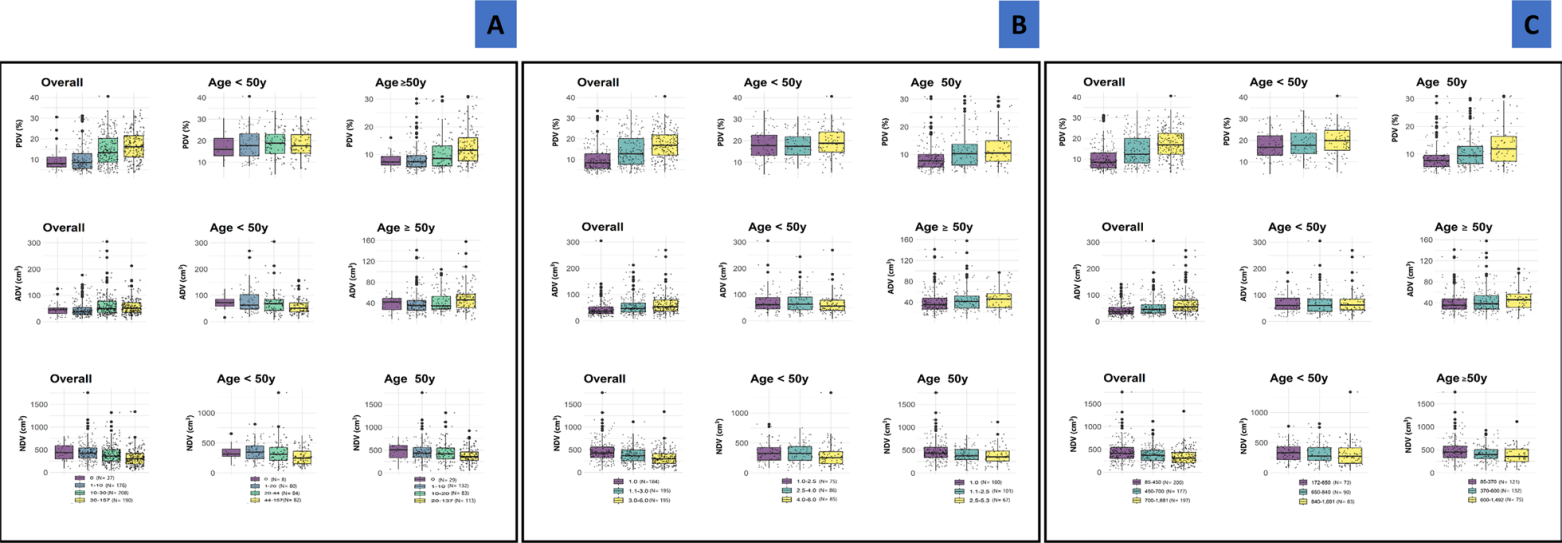
Distribution of quantitative mammographic density measures by terminal ductal lobular unit (TDLU) measurement categories. **A** TDLU count per 100 mm^2^, **B** median acini count per TDLU, and **C** median TDLU span. For women with observable TDLUs, overall and age-group specific tertiles of median TDLU span and median acini count/TDLU were calculated

Overall and among older women, the three TDLU measures were positively correlated with PDV and ADV and negatively correlated with NDV (Fig. 1). The TDLU-MD correlation was stronger for PDV (r^2^ = 0.45–0.49, *p* < 0.0001) than for ADV (r^2^ = 0.22–0.27, *p* < 0.0001). These patterns are also shown in Fig. 2, which shows that PDV and, to a lesser extent, ADV, increased while NDV decreased with increasing TDLU measures among all and older women. These relationships were attenuated among younger women. Particularly, ADV decreased with increasing TDLU measures especially for TDLU count among younger women.

We then used generalized linear regression models to assess the associations between TDLU involution and MD measures after adjusting for potential confounders including age, BMI, parity, age at menarche, TBV (for ADV), and breast cancer subtype (Table 2). Among all women, PDV was positively associated with TDLU count (p_trend_ < 0.0001), TDLU span (p_trend_ < 0.0001), and acini count/TDLU (p_trend_ = 0.0005). These associations remained significant after correcting for multiple corrections. PDV was 28% higher among women with ≥ 30 TDLU count/100m^2^ compared to those with 0 TDLU count/100 mm^2^ (95% CI 1.08–1.51). Similarly, PDV was 23% and 22% higher, respectively, among women in the highest vs. the lowest tertile of median TDLU span (95% CI 1.11–1.37) and median acini count/TDLU (95% CI 1.09–1.37, Table 2). Similar associations were found between PDV and all TDLU measures among women ≥ 50 years. However, among women < 50 years there was no statistically significant association between PDV and any of the three TDLU measures. Similar trend was observed for ADV after the adjustment of TBV, although the associations for ADV were in general weaker than those for PDV (Table 2).

**Table 2:** Association between TDLU measures and mammographic density variables

		Percent Dense Volume^a^					Absolute Dense Volume^a,b^					Non Dense Volume^a^				
TDLU Measure^c^	N = 611	Mean^a^	exp(beta)^a^	95% CI^a^		P Value^a^	Mean^a,b^	Exp (beta)^a,b^	95% CI^a,b^		P Value^a,b^	Mean^a^	exp(beta)^a^	95% CI^a^		P Value^a^
*Overall*																
TDLU count/100 mm^2^																
0	37	9.95	Reference				39.50	Reference				357.15	Reference			
1–< 10	176	10.57	1.06	0.90	1.25	0.4719	41.33	1.05	0.90	1.21	0.5482	339.58	0.95	0.78	1.16	0.6137
10–< 30	208	12.50	1.26	1.07	1.48	**0.0060**	47.36	1.20	1.03	1.39	**0.0160**	304.47	0.85	0.70	1.04	**0.1086**
30–157	190	12.72	1.28	1.08	1.51	**0.0043**	45.07	1.14	0.98	1.33	0.0931	254.78	0.71	0.58	0.87	**0.0010**
Trend^d^		** < 0.0001**^**e**^					**0.0246**					** < 0.0001**^**e**^				

	N = 574	Percent dense volume^a^					Absolute dense volume^a,b^					Non dense volume^a^				
Median TDLU span (µm)																
85–< 450	200	10.30	Reference				39.71	Reference				332.84	Reference			
450–< 700	177	12.45	1.21	1.10	1.33	** < 0.0001**^**e**^	46.49	1.17	1.07	1.28	**0.0003**^**e**^	298.16	0.90	0.80	1.01	0.0614
700–1691	197	12.67	1.23	1.11	1.37	**0.0001**^**e**^	46.17	1.16	1.06	1.28	**0.0020**	278.03	0.84	0.74	0.95	**0.0057**
Trend^d^		** < .0001**^**e**^					**0.0014**					**0.0053**				
Median acini count/TDLU																
1	184	10.25	Reference				40.31	Reference				350.94	Reference			
1.1–< 3	195	12.18	1.19	1.08	1.31	**0.0004**^**e**^	45.61	1.13	1.04	1.24	**0.0057**	300.7	0.86	0.76	0.96	**0.0088**
3–6	195	12.52	1.22	1.09	1.37	**0.0005**^**e**^	44.50	1.10	1.00	1.22	0.0600	262.45	0.75	0.65	0.86	** < 0.0001**^**e**^
Trend^d^		**0.0005**^**e**^					0.0577					** < 0.0001**^**e**^				

		Percent Dense Volume^a^					Absolute Dense Volume^a,b^					Non Dense Volume^a^				
TDLU Measure^c^	N = 254	Mean^a^	exp(beta)^a^	95% CI^a^		P Value^a^	Mean^a,b^	exp(beta)^a,b^	95% CI^a,b^		P Value^a,b^	Mean^a^	exp(beta)^a^	95% CI^a^		P Value^a^
*Women* < *50 years*																
TDLU count/100 mm^2^																
0	8	16.65	Reference				57.56	Reference				287.18	Reference			
1–≤ 20	80	17.60	1.06	0.80	1.39	0.6908	60.88	1.06	0.81	1.38	0.6762	284.11	0.99	0.67	1.46	0.9567
21–≤ 44	84	18.60	1.12	0.85	1.47	0.4247	62.13	1.08	0.83	1.40	0.5667	237.27	0.83	0.56	1.21	0.3317
45–157	82	18.15	1.09	0.83	1.43	0.5382	58.67	1.02	0.78	1.33	0.8876	204.04	0.71	0.48	1.05	0.0850
Trend^d^		0.4682					0.6911					** < .0001**^**e**^				

	N = 246	Percent dense volume^a^					Absolute dense volume^a,b^					Non dense volume^a^				
Median TDLU span (µm)																
172–< 650	73	17.07	Reference				57.96	Reference				265.96	Reference			
650–< 840	90	18.26	1.07	0.95	1.21	0.2794	60.59	1.05	0.93	1.18	0.4579	233.83	0.88	0.73	1.05	0.1591
840–≤ 1691	83	19.05	1.12	0.99	1.26	0.0802	63.14	1.09	0.97	1.23	0.1541	230.67	0.87	0.72	1.04	0.1205
Trend^d^		0.0825					0.1537					0.1334				
Median acini count/TDLU																
1–< 2.5	75	17.49	Reference				59.59	Reference				268.51	Reference			
2.5–< 4	86	17.89	1.02	0.91	1.15	0.7129	60.46	1.01	0.91	1.14	0.8036	258.38	1.00	0.81	1.1437	0.6625
4–6	85	18.68	1.07	0.95	1.21	0.2913	61.03	1.02	0.91	1.15	0.6898	216.71	0.99	0.68	0.9628	**0.0173**
Trend^d^		0.2879					0.6906					**0.0160**				

		Percent dense volume^a^					Absolute dense volume^a,b^					Non dense volume^a^				
TDLU Measure^c^	N = 357	Mean^a^	exp(beta)^a^	95% CI^a^		P Value^a^	Mean^a,b^	exp(beta)^a,b^	95% CI^a,b^		P Value^a,b^	Mean^a^	exp(beta)^a^	95% CI^a^		P Value^a^
*Women* ≥ *50 years*																
TDLU count/100 mm^2^																
0	29	7.32	Reference				32.18	Reference				414.64	Reference			
1–≤ 9	132	7.91	1.08	0.88	1.33	0.4578	34.09	1.06	0.89	1.26	0.5149	393.05	0.95	0.76	1.18	0.6363
10–≤ 19	83	8.99	1.23	0.99	1.52	0.0611	37.59	1.17	0.97	1.40	0.0954	365.02	0.88	0.70	1.11	0.2840
20–137	113	10.84	1.48	1.20	1.83	**0.0003**	41.79	1.30	1.08	1.56	**0.0051**	305.44	0.74	0.58	0.93	**0.0099**
Trend^d^		** < 0.0001**^**e**^					**0.0117**					**0.0005**^**e**^				

	N = 328	Percent dense volume^a^					Absolute dense volume^a,b^					Non dense volume^a^				
Median TDLU span (µm)																
85–< 370	121	6.83	Reference				30.63	Reference				433.67	Reference			
370–< 600	132	7.89	1.16	1.02	1.31	**0.0253**	33.32	1.09	0.98	1.21	0.1231	382.78	0.88	0.77	1.01	0.0695
600–1492	75	9.05	1.33	1.14	1.54	**0.0002**	36.02	1.18	1.03	1.34	**0.0131**	335.31	0.77	0.66	0.91	**0.0016**
Trend^d^		**0.0002**^**e**^					0.3857					**0.0015**				
Median acini count/TDLU																
1	160	6.97	Reference				31.06	Reference				427.30	Reference			
1.1–< 2.5	101	8.37	1.20	1.06	1.36	**0.0049**	34.58	1.11	1.00	1.24	0.0508	363.37	0.85	0.74	0.97	**0.0189**
2.5–5.3	67	8.72	1.25	1.07	1.46	**0.0040**	34.93	1.12	0.99	1.28	0.0760	341.81	0.80	0.68	0.94	**0.0070**
Trend^d^		**0.0012**					**0.0414**					**0.0031**				

Significant results based on *p* < 0.05

^a^Continuous percent dense volume, absolute dense volumes and non-dense volume were log transformed in the generalized linear analysis. The means, β coefficients and their corresponding 95% CI were back transformed for ease of intepretation. Models were adjusted for age at diagnosis, BMI, parity, age at menarche and breast cancer subtype

^b^Models for absolute dense volume were further adjusted for total breast volume

^c^For women with observable TDLUs, overall and age-group specific tertiles of median TDLU span and median acini count/TDLU were calculated

^d^*p* values were obtained by treating the categorical TDLU variables as ordinal variables in trend analyses

^e^Result that maintained statistical significance after accounting for multiple testings

Overall and among women ≥ 50 years, NDV was negatively associated with TDLU count, TDLU span and acini count/TDLU (Table 2). Overall, NDV was 29%, 16%, and 25%, respectively, lower among women with the greatest TDLU count (95% CI 0.58–0.87), median TDLU span (95% CI 0.74–0.95), and median acini count/TDLU (95% CI 0.65–0.86) compared with women with the lowest TDLU measures. Among women < 50 years, only TDLU count and acini count/TDLU were associated with NDV.

In separate analyses using GEE models to model TDLU span as the outcome variable and MD measures as explanatory variables, which allowed for analysis of all TDLU span data points, we observed similar associations to those seen in the primary analyses for all MD measures (Table 3).

**Table 3:** Associations between TDLU span and MD phenotypes

	Beta^a^	95% CI^a^		*p* value^a^
*All women*				
PDV	5.47	2.26	8.68	**0.0008**
ADV^b^	0.74	0.13	1.35	**0.0178**
NDV	− 0.14	− 0.25	− 0.03	**0.0096**
*Women < 50 years*				
PDV	3.77	− 1.14	8.68	0.1322
ADV^b^	0.36	− 0.50	1.21	0.4133
NDV	− 0.11	− 0.30	0.08	0.2623
*Women ≥ 50 years*				
PDV	7.39	3.31	11.48	**0.0004**
ADV^b^	1.35	0.19	2.51	**0.0222**
NDV	− 0.17	− 0.29	− 0.05	**0.0055**

Significant results based on *p* < 0.05

^a^Results from generalized estimating equations (GEE) models when accounting for the lack of independence of repeated TDLU span measures; TDLU span was treated as the outcome and percent dense volume (PDV), absolute dense volume (ADV), non-dense volume (NDV) were the explanatory variables. Models were further adjusted for age, body mass index, age at mernarche, parity and tumor subtype

^b^Models for ADV were further adjusted for total breast volume

### Associations between TDLU measures and mammographic density variables stratified by breast cancer subtype

**Fig. 3:**
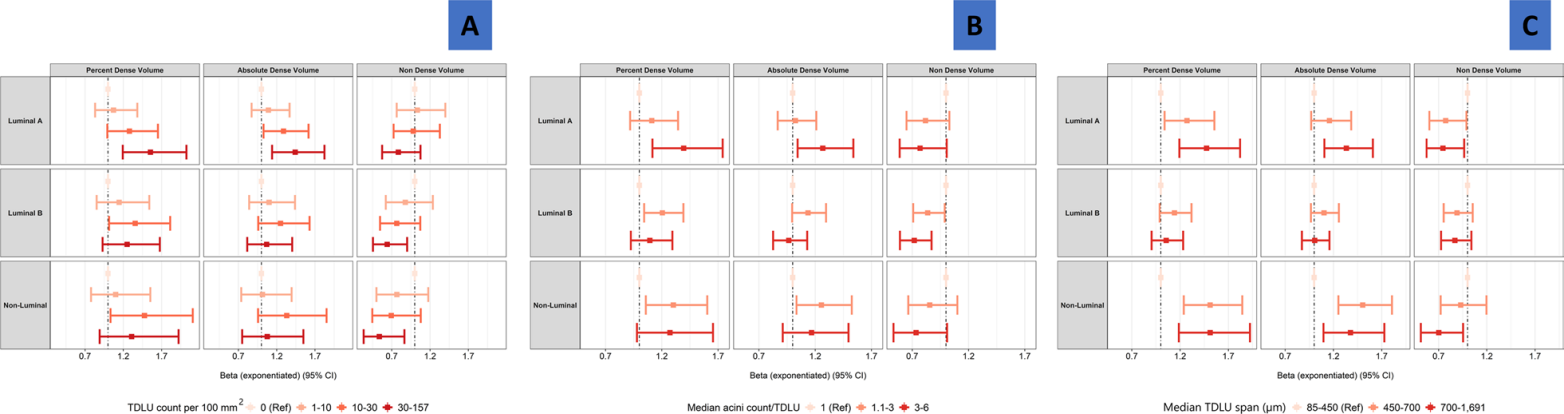
Associations between terminal ductal lobular unit (TDLU) measures and mammographic density variables stratified by breast cancer subtype. Continuous percent dense volume (PDV), absolute dense volume (ADV) and non-dense volume (NDV) were log transformed in the analysis. The β coefficients and their 95% confidence intervals were back transformed for ease of interpretation. Models were adjusted for age at diagnosis, BMI, parity, age at menarche. Models for absolute dense volume were further adjusted for total breast volume

Since both MD and TDLU measures appeared to vary by breast cancer subtype in our analysis, we further addressed whether the associations between TDLU and MD measures varied by breast cancer subtypes by conducting multivariable-adjusted linear regression analyses in luminal A, luminal B (combining luminal B/HER2− and luminal B/HER2+), and non-luminal (combining HER2-enriched and TN) subtypes separately (Fig. 3). Overall, associations between TDLU and MD measures showed similar patterns across molecular subtypes, although the ordinal trend for PDV with TDLU span (P_het_ = 0.0409) and ADV with TDLU count (P_het_ = 0.0173) were stronger in luminal A than in luminal B patients (see Supplementary Table 4).

## Discussion

Our study findings suggest a complex relationship between MD and TDLU involution which depends on age and MD measures. After adjusting for covariates, we found that women with higher PDV had higher TDLU count, larger median TDLU span, and more acini count per TDLU reflecting an inverse relationship between PDV and TDLU involution. There was a positive relationship between NDV and TDLU involution, whereas ADV in general was not associated with TDLU involution. However, after further adjustment of TBV, the associations for ADV were similar to those for PDV, albeit weaker with ADV. In stratified analyses, we found that the associations for PDV and NDV with TDLU measures were more pronounced among older women compared to younger women. In addition, we generally observed stronger associations between PDV and TDLU measures in patients with luminal A tumors than other subtypes. Consistent with previous studies of BBD [3, 16, 17] and breast cancer patients [18], we showed an inverse relationship between PDV and TDLU involution. Our study extends previous findings by using quantitative objective measures of TDLU and MD in a large cohort of Chinese breast cancer patients and stratifying by breast cancer subtype.

Consistent with findings from previous studies among women with BBD [3, 17], we found stronger associations of TDLU measures with PDV than with ADV. The differences may reflect the distinct tissue composition estimated by different density measures. While ADV reflects the absolute abundance of stromal and epithelial (i.e., fibroglandular) tissue, PDV measures the proportion of total breast tissue that is fibroglandular tissue relative to adipose tissue (NDV). Indeed, after the adjustment of the total breast volume, we observed similar MD-TDLU associations for PDV and ADV. Previous studies reported that percent density, dense area/volume, and non-dense area/volume were independently associated with breast cancer risk [23, 24], suggesting that breast epithelium, stroma, and adipose tissue may influence breast cancer development and progression through distinct mechanisms [25]. Data from a recent study in BBD patients demonstrated that percent density and epithelium-to-stroma proportion, a histologic metric of the proportion of fibroglandular tissue that is epithelium relative to stroma, were independently associated with breast cancer risk, further highlighting the complex role of breast tissue composition in breast cancer development [26].

In line with this, well-established breast cancer risk factors also showed differential associations with different breast tissue composition. For example, in studies of healthy women and women with BBD, parity was associated with higher amount of epithelium/TDLUs but lower amount of stroma [27, 28]. Our results showing distinct associations of MD and TDLU measures with parity among breast cancer patients are largely in line with these findings. Additionally, we showed that the associations between parity and breast tissue composition measures varied by age. While parity was positively correlated with TDLU number and size among younger women, it was negatively correlated with these features among older women. Further, the inverse associations between parity and ADV/PDV were weaker among younger than older women. Our observed results may be explained, at least in part, by tissue changes that occur because of post-lactational and age-related lobular involution, which are distinct processes [6]. While lobular involution is related to aging of the mammary gland, post-lactational involution is the process by which breast tissues return to their pre-partum state through apoptosis and tissue remodeling [29]. The positive associations between parity and TDLU measures among younger women may be explained in part by parity-related epithelial hyperplasia, while the weak associations with ADV/PDV in this age group may be explained by the fact that parity-associated epithelial hyperplasia is accompanied by concomitant reduction in stroma [28], which is a major component of ADV/PDV [30].

Age-related TDLU involution is a dynamic process. The relationships between TDLU and MD measures depend on whether TDLUs are replaced with dense stroma or non-dense fat, a process that is determined by age as well as other factors that affect tissue remodeling processes such as those affecting extracellular matrix organization, adipocyte differentiation, and immune cell composition [31]. Pregnancy-related involution may add another layer of complexity of tissue composition among younger/pre-menopausal women, which in combination may explain our finding that the inverse relationship between PDV and TDLU involution was more prominent among older than younger women [3]. Our findings are, however, in contrast to those in the study among BBD patients in Vermont, USA by Gierach et al. [17], in which an inverse relationship between TDLU involution and MD was observed among pre-menopausal women, but not among post-menopausal women. The mixed results may be attributed to heterogeneous study populations, sample size, risk factors, mode of detection, and assessment of TDLU and MD.

We previously found that higher MD was associated with HER2-enriched and luminal B breast subtypes among Chinese breast cancer patients [8, 20], while reduced TDLU involution was associated with TNBC/basal-like breast subtypes among European, Black, and Chinese women [10–12]. Here, we found that the associations between TDLU involution and MD measures mostly showed similar patterns across luminal A, luminal B and non-luminal breast cancer subtypes. However, the associations for PDV with TDLU span and ADV with TDLU count were stronger among patients with luminal A tumors compared with luminal B tumors. Molecular mechanisms underlying the differential subtype association remain unclear and it is possible that the observed heterogeneity/lack of heterogeneity is due to limited statistical power. Future studies integrating molecular data with radiologic and histopathologic features are needed to better understand the biological pathways underlying these processes and their interactions.

The strengths of our study include access to a large dataset of uniquely unscreened Asian breast cancer patients with well-annotated pathology (including breast cancer subtype) and clinical data, the collection of key exposures related to TDLU/MD, and quantitative measures for both TDLU and MD variables. However, our study has several limitations. Our study population consists of Chinese breast cancer patients, and TDLU assessment was based on adjacent normal tissue. As a result, the generalization of our findings to healthy women or other populations may be limited. In addition, since the patients were selected from a single hospital, which is one of the best oncology hospitals in China, our patient population is also not representative of the general breast cancer patient population in China. Furthermore, the raw images or Volpara density maps were not available for tissue-based regional analysis, and by virtue of restricting this analysis to the contralateral breast, the subject-level density results do not reflect densities at regions where TDLUs were sampled. However, a sensitivity analysis using MD measures from the diseased breast showed no changes in our findings. In addition, we did not have information on use of oral contraceptives or hormone-replacement therapy. However, the use of hormone-replacement therapy is low among Chinese women therefore it most likely would not have impacted our results. Although this study represents one of the largest conducted on this research topic, our sample size was still limited, and we had to collapse certain subtype categories (e.g., combining luminal B/HER2− and luminal B/HER2+ into a single luminal B group, and merging HER2-enriched with TNBC into a non-luminal group). This categorization might have potentially obscured some associations especially as our previous studies conducted in breast cancer patients from the same hospital have found differential associations with subtypes for HER2 (associated with higher MD compared to luminal A [8, 9]) and TNBC (associated with lower TDLU involution compared to luminal A [10]).

## Conclusions

By utilizing quantitative measures of MD, we have corroborated and extended previous findings of the inverse associations between TDLU involution and PDV/ADV in women of predominantly European ancestry to women of Asian ancestry. Furthermore, we demonstrated that these inverse associations were more pronounced in older women and those with luminal A tumors, compared to younger women or women with luminal tumors. Our results provide additional evidence to the evolving understanding of the dynamic interplay between MD and TDLU involution, specifically in an Asian population characterized by notable disparities in breast cancer incidence, risk factor profiles, and MD distributions compared to European populations.

### Supplementary Information

Supplementary Material 1: Table 1. Comparison of selected cases for the current study with those not selected.

Supplementary Material 2: Table 2. Intra variability of TDLU measures.

Supplementary Material 3: Table 3. Patient characteristics and distribution of terminal ductal lobular involution unit (TDLU) measurements.

Supplementary Material 4: Table 4. Heterogeneity of mammographic density and terminal ductal lobular involution unit (TDLU) associations across breast cancer subtypes.

## Data Availability

The datasets used and/or analyzed during the current study are available from the corresponding author on reasonable request.

## Abbreviations

ADV: Absolute dense volume
BBD: Benign breast disease
BI-RADS: The Breast Imaging Reporting and Data System
BMI: Body mass index
CHCAMS: The Cancer Hospital, Chinese Academy of Medical Sciences
CI: Confidence interval
GEE: Generalized estimating equations
MD: Mammographic density
NDV: Non-dense volume
PDV: Percent dense volume
SD: Standard deviation
TBV: Total breast volume
TNBC: Triple negative breast cancer
TDLU: Terminal duct lobular unit
